# Copper-Induced Strengthening in 0.2 C Bainite Steel

**DOI:** 10.3390/ma14081962

**Published:** 2021-04-14

**Authors:** Jaromir Dlouhy, Pavel Podany, Jan Dzugan

**Affiliations:** COMTES FHT a.s., Prumyslova 995, 334-41 Dobrany, Czech Republic; pavel.podany@comtesfht.cz (P.P.); jan.dzugan@comtesfht.cz (J.D.)

**Keywords:** bainite, copper precipitation, manganese, hole expansion test

## Abstract

Bainitic steels were the focus of this study. These steels have the potential to obtain a good combination of strength, ductility, and edge stretchability, which is a very desirable characteristic in the automotive industry. Cu precipitation potential was investigated during prolonged isothermal bainitization treatment. Precipitation strengthening and ductility were measured using a tensile test, and edge stretchability was measured using a hole expansion test. The microstructure was characterized by high-resolution scanning electron microscopy and an electron backscattered diffraction. Lower bainite was obtained by austenitization treatment and subsequent immersion into a salt bath at 400 °C. Cu precipitation occurred after 120 min of holding in the bath and enhanced the yield stress of the Cu-alloyed steel by 120 MPa as compared with a reference steel without Cu. The strengthening did not affect ductility and decreased the edge stretchability by 10%. Steels with different Mn contents were examined. It was found that the enhancement of Mn content from 1 to 2 wt.% did not boost Cu strengthening ability. This result showed that the presence of Mn did not cause an Mn-Cu precipitation strengthening synergy, observed previously during martensite tempering procedure.

## 1. Introduction

High strength steels are a group of materials that have received significant attention in the field of material research. Advancements in rolling technology have enabled the production of sheets with outstanding combinations of strength and ductility [1,2]. These sheet materials are extensively used, mainly in the automotive industry. Car developers relentlessly seek ways to save weight in their product, therefore there is a need for better technological and better mechanical properties.

The term “better mechanical properties” initially focused mainly on the strength-ductility trade-off. Dual-phase, TRIP (transformation-induced plasticity), and complex phase steels have been developed and gradually introduced for industrial use. Their strength-ductility combination has exceeded conventional ferritic or martensitic steels. There were two main sources of this excellency, i.e., grain refinement and a combination of soft and hard phase in the structure.

Subsequently, the focus has been on another material parameter, i.e., edge stretchability [3] which is a technological property crucial for cold forming production lines. Multiphase steels generally suffer from poor edge stretchability due to soft and hard phase coexistence in their structure [4,5,6]. Monophase steels generally exhibit better values [7]. Even the presence of retained austenite has been proven to be detrimental to the fracture toughness of high strength steel sheets [8,9,10]. This is important for the group of bainitic steels because a carbide-free bainite (i.e., mixture of bainitic ferrite and retained austenite) is intensively studied as a promising structure of high strength steels [11,12,13]. A study focused on the “ferrite-carbide” bainite structure as a structure with potential to deliver a good combination of strength, plasticity [14], and also edge stretchability.

The aim of this study is to describe the influence of Cu content on the mechanical properties of bainitic 0.2 wt.% C steel. The concept of a low-carbon, low-alloyed steel meets the criteria for a weldable low-cost steel suitable for mass production. Isothermal bainitic quenching can be achieved relatively easily in the form of hot rolling, partial cooling, and coiling of a steel strip [2]. Cu alloying has been extensively studied due to the Cu precipitation phenomena [15,16,17]. Cu is significantly more soluble in the austenite than in the ferrite. This provides an opportunity to dissolve Cu in a solid solution at a high temperature, cool the material to form supersaturated Cu solid solution in ferrite, and then let the Cu precipitate from supersaturated solution at a certain temperature (usually in range from 400 to 600 °C). Cu precipitates with several nanometers in diameter significantly strengthen the ferrite matrix [18].

We also address the influence of Mn content. Mn was reported to be an active element in Cu precipitation phenomena by segregating itself on the Cu precipitate-ferrite matrix interphase [19,20]. A previous study by [21] demonstrated significant Mn influence on Cu precipitation during martensite tempering. Enhanced Mn content significantly improved Cu precipitation strengthening of the experimental steels.

## 2. Materials and Methods

Four steels with a different chemical composition were casted for the experiment. The steels differed in Cu and Mn contents, as can be seen in the Table 1. The melts were produced in a vacuum induction furnace. Gross melt weight was 500 kg, and the melts were casted into a round ingot mold with 220 mm diameter. Ingots were forged into billets and the billets underwent hot rolling to a thickness of 5 mm and cold rolling to the final thickness of 1.5 mm and width 300 mm. The details of the steel casting and forming are provided in [21].

The sheets were cut into samples for tensile testing and a hole expansion test (HET). The samples for tensile testing were 230 mm long and 35 mm wide (longer direction being the rolling direction), the samples for HET were circular discs with 130 mm diameter.

**Figure 1:**
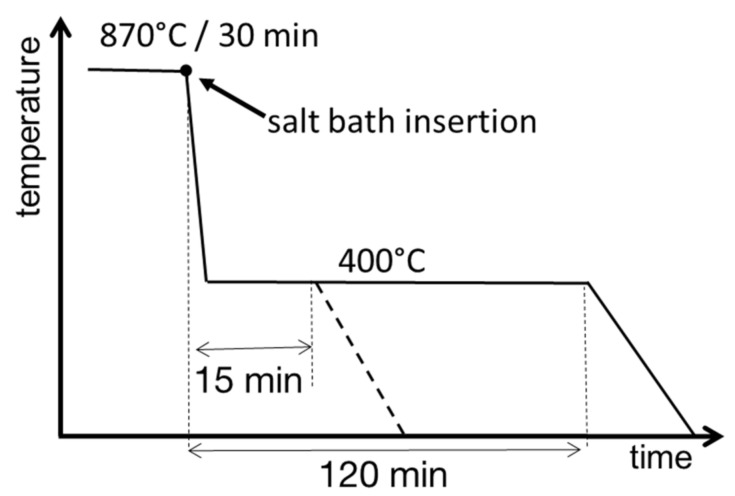
Schematic of the heat treatment.

All samples underwent heat treatment according to the schematic in Figure 1, which included austenitization and bainitization treatments in a salt bath. The samples were austenitized in an atmospheric electric furnace (BVD PECE spol. s.r.o., Podlesí, Czech Republic) at 870 °C for 30 min. They were protected from scaling and decarburization by protective coating Tinderex (Rembrandtin Lack GmbH Nfg. KG, Wien, Austria). Bainitization was performed by inserting the samples into a salt bath Pragokor AS140 (Pragochema spol. s.r.o., Prague, Czech Republic), preheated to 400 °C. The samples stayed in the salt bath for 15 and 120 min. They were pulled out afterwards and left to cool in air.

**Figure 2:**
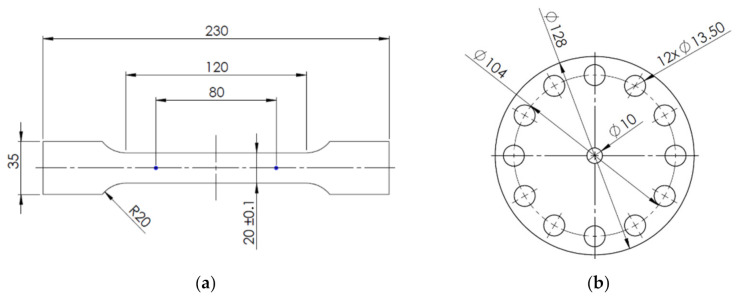
Specimen drawings (all dimensions in mm). (**a**) Tensile-test specimen; (**b**) HET specimen.

Specimens for mechanical testing were manufactured from the heat-treated samples. Tensile testing specimens were produced by milling the specimens’ contour. HET specimens were prepared by punching a 10 mm hole in the center of the disk (Figure 2).

Quasistatic tensile tests were performed at room temperature according to the standard EN ISO 6892-1 with a displacement control at the indicated crosshead speed of 1 mm/min, using a Zwick Z250 testing machine with a 250 kN capacity (ZwickRoell GmbH & Co. KG, Ulm, Germany). Three specimens were tested for each material and length of the bainitization treatment. Yield stress (YS) was determined as a 0.2% proof stress. The following additional parameters were determined: engineering ultimate tensile stress (UTS), homogeneous plastic elongation (Ag) determined at maximal strength, elongation after fracture at initial measured length 80 mm (A80), and reduction of area (ROA). Elongation was measured by a strain-gauge-based clip-on extensometer.

HET was performed according to standard ISO 16630. A center 10 mm punched hole was stretched by conical punch with a 60° angle. The punch moved with speed 1 mm/sec. The test was recorded by a digital image correlation (DIC) Aramis system. The record was used to determine the exact moment of the hole edge cracking and the hole expansion was measured at that moment. The hole expansion ratio (HER) was calculated according to the standard as follows:

(1) $$\mathrm{HER}\left[\%\right]=\frac{D_{e}-D_{0}}{D_{0}}\times 100,$$

where *D*_0_ is the initial hole diameter and *D_e_* is the expanded hole diameter at the moment of the hole edge cracking. Six samples were tested for each material and bainitization time.

The microstructure was characterized by high-resolution scanning electron microscopy (SEM) and electron backscattered diffraction (EBSD). Specimens were cut out of the tensile specimen heads in a longitudinal (rolling) direction. They were embedded into resin, mechanically ground, and polished. The final polishing was done with colloidal silica solution with mean grain size 50 nm.

The specimens were observed by SEM JEOL IT-500 HR at 15 kV acceleration voltage. The EBSD analysis was performed at 20 kV acceleration voltage. Detailed maps were acquired with step size 0.05 µm and size 60 × 45 µm. Grain size (GS), grain aspect ratio (AR), and kernel average misorientation (KAM) values were extracted from the EBSD data.

## 3. Results

Tensile testing revealed the influence of chemical composition and the duration of salt bath treatment on the mechanical properties (Table 2).

The 15-min bainitization treatment resulted in materials with almost identical ductility, except slightly lower A_g_ content of the 0C steel as compared with the Cu-alloyed ones. The difference in alloying was significantly noticeable in the YS and UTS values. 1Cu and 1.5Cu steels exhibited YS and UTS values higher by about 60 MPa as compared with the reference 0Cu steel, and 1.5Cu2Mn steel differed by about 45 MPa in these values.

The treatment of 120 min of holding in the salt bath did not noticeably change the mechanical properties of the 0Cu steel as compared with that of the 15-min holding treatment. However, Cu-alloyed steels enhanced their YS and UTS values significantly without any loss of ductility.

The HER values exhibited large scattering among six tested specimens. No trend can be assumed from the 15-min treated samples. There was a reduction in the HER regarding Cu-alloyed steels treated for 120 min as compared with 0Cu steel.

**Figure 3:**
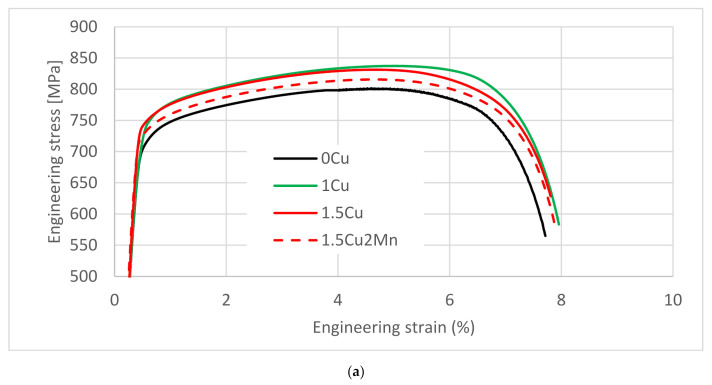

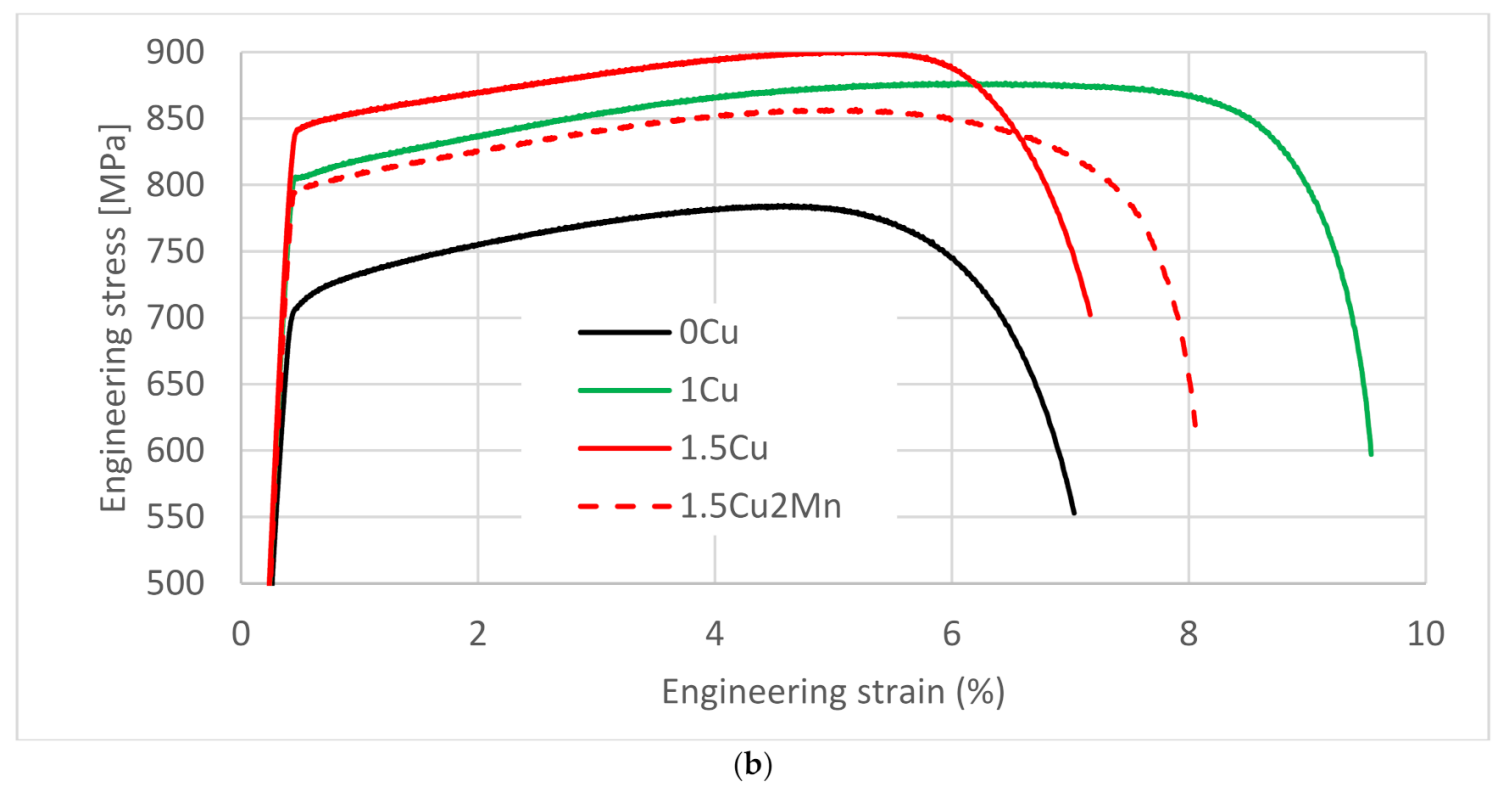
Stress-strain curves of the tensile test specimens. (**a**) 15-min bainitization; (**b**) 120-min bainitization.

The stress-strain curves (Figure 3) were analyzed to obtain values of the engineering stress drop (decreased UTS) from maximal value to the final force and toughness in a quasistatic tension (Table 3). The toughness was determined as the area under the stress-strain curve (AuC), thus, expressed in MPa. The AuC followed the trend of Cu-related strengthening. Higher YS and UTS generally led to higher AuC because of comparable ductility of all materials and the same general shape of the stress-strain curves. The decrease in the UTS values did not follow any trend for the 15-min bainitization treatment. There was a difference in the case of the 12-min bainitization treatment, where 1.5Cu and 1.5Cu2Mn steels exhibited lower values of UTS than the 0Cu and 1Cu steels.

**Figure 4:**
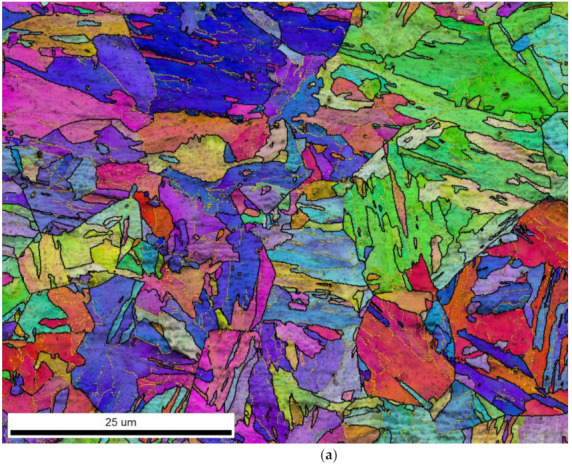

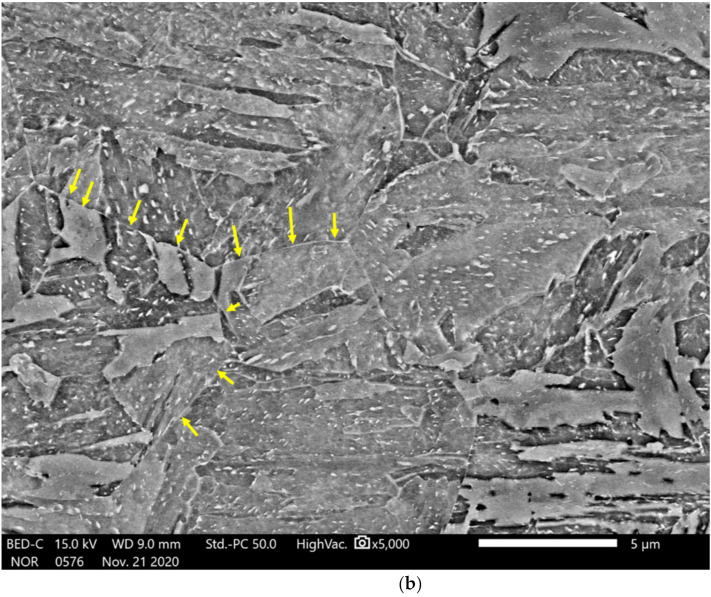
The 1Cu 15-min treated sample. (**a**) Electron backscattered diffraction (EBSD) map colored by orientation. Black lines show high angle boundaries (misorientation angle >15°). Thin yellow lines show low angle boundaries (misorientation angle >2° and <15°); (**b**) SEM image of the microstructure showing both the ferrite matrix structure and carbide distribution (bright particles). Arrows show prior austenite grain boundaries.

The microstructure of all examined samples was composed of bainite. Figure 4 shows the microstructure overview of the 1Cu 15-min treated sample. The EBSD map displays the structure of the ferrite matrix. The plate-like appearance is typical for ferrite formed by shear transformation. However, individual ferrite plates are not all clearly resolved and are arranged in stacks of similarly oriented plates.

The SEM micrograph also shows the matrix structure using gray shading and bright lines for the grain boundaries. The SEM micrographs were scanned by backscattered electron (BSE) detector to gain as much grain contrast as possible. The SEM observation further revealed the distribution of the carbides in the structure. There did not seem to be any retained austenite in the structure. Generally, the carbides were dispersed homogeneously in the matrix. Prior austenite grain boundaries were visible. There was no difference in general microstructure appearance among all samples. The presented overview of the microstructure for the 1Cu 15-min treated sample (Figure 3) can be taken as a representative one for all other experimental steels. Quantitative features of the bainite structure regarding grain size and shape are provided further in the discussion for all samples after the 15-minute bainitization treatment.

**Figure 5:**
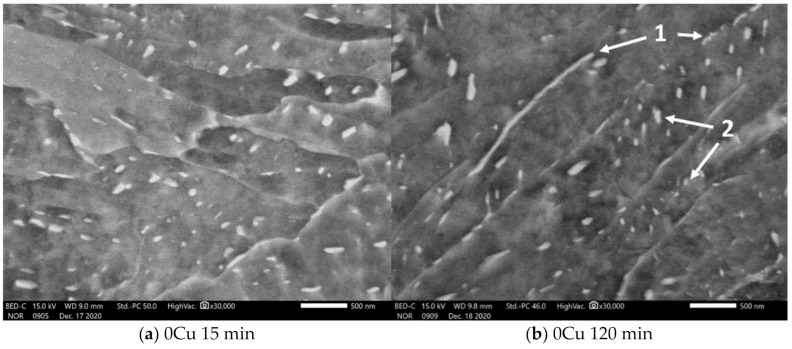

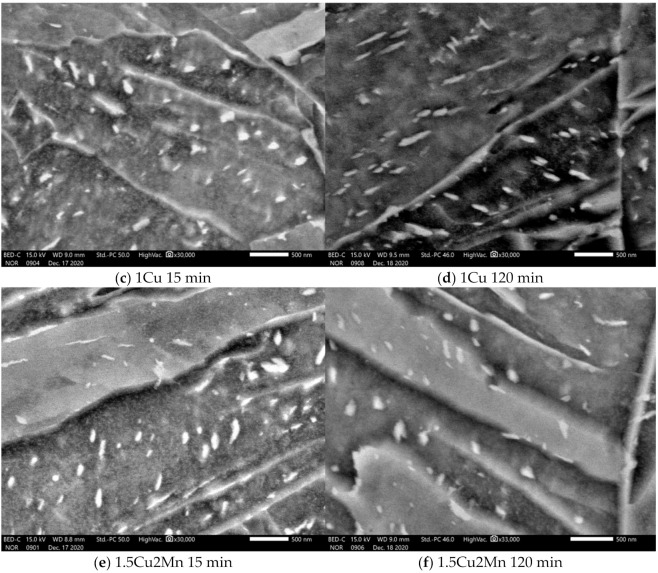
Micrographs of the experimental steels after 15 min and 120 min of holding at 400 °C. The structure is composed of a lower bainite. The plate-like structure of the ferrite matrix is visible. Distinctive features of the carbides are marked in panel (**b**): carbides (bright particles) are present both on the plate boundaries (1) and in the interior of the ferrite plates (2). There is no feature in the microstructure on this scale to distinguish one sample from another, in terms of carbide distribution, size, shape, and ferrite crystals morphology.

Next, we described the detailed micrographs of the microstructures. Figure 5 demonstrates basic features of the microstructure composed of lower bainite [22] (pp. 66–68). The matrix is formed by plate-like ferrite crystals. Carbides are present both on the plate boundaries and in their interior. The shape of the carbides is generally elongated. Boundary carbides have grown along the grain boundary. Carbides in the plate’s interior are elongated and parallel to each other.

There was no observable effect of the salt bath holding time on the carbide size, distribution, or morphology.

**Figure 6:**
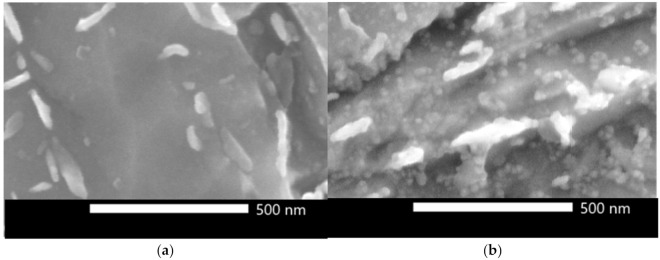
Detailed secondary electron micrographs of the (**a**) 0Cu and (**b**) 1.5Cu steel after 120 min of holding at 400 °C. Bright elongated carbides (up to 300 nm long) are visible in both steels. There are also globular particles with diameters below 30 nm visible in the 1.5Cu steel matrix.

Figure 6 compares 0Cu and 1.5Cu microstructures at a submicron scale. Globular particles up to 30 nm in diameter were observed in the 1.5Cu steel. They were dispersed homogeneously in the ferrite matrix. No such particles were observed in the 0Cu steel.

## 4. Discussion

Differences in YS were observed among experimental steels. The YS value depends on several strengthening factors [22] as follows:

- Lattice slip stress;
- Grain boundary strengthening;
- Dislocation density;
- Solid solution strengthening;
- Precipitation strengthening.

**Figure 7:**
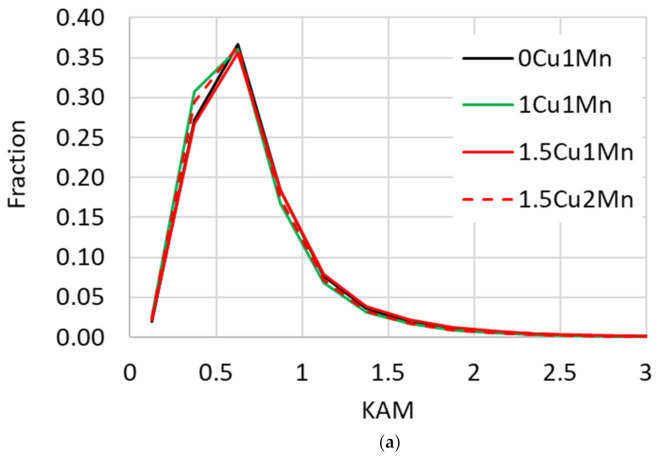

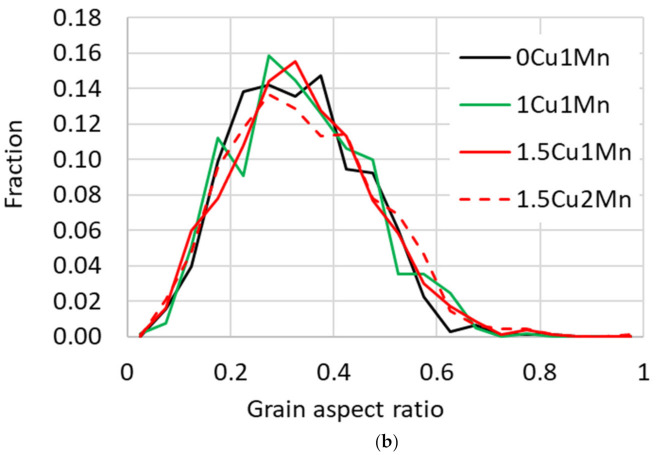
The EBSD map analysis of the 15-min treated samples. (**a**) Kernel average misorientation (KAM) distribution (KAM was measured for each point in the map); (**b**) distribution of grain aspect ratio. Grains were defined as areas enclosed by boundaries with >2° misorientation.

The lattice slip stress can be assumed to be equal for all experimental steels (being a constant of the ferrite lattice). Grain boundary strengthening depends on the grain size and in the case of non-equiaxial grains also on grain shape. These parameters were both determined from the EBSD maps. Grains were defined as areas surrounded by boundaries with a misorientation of 2°. This lower limit was usually considered to be a low-angle boundary misorientation limit (high-angle one being usually set on 15°). Therefore, the grain analysis took both high- and low-angle boundaries into account and effectively displayed values for the subgrain structure. This approach was chosen due to the nature of the bainitic structure composed of elongated ferrite crystals separated often only by low-angle boundaries. Table 4 shows the average grain size and Figure 7b shows the distribution of grain aspect ratio. These parameters can both be considered to be identical for all experimental steels.

The EBSD analysis included computation of the kernel average misorientation (KAM) which was computed for each point on the EBSD map. Its value is an average misorientation between a given point and each of its neighbors. It gives an estimate of the lattice curvature, and therefore also an estimate of the dislocation density at a given point [23]. Figure 7a shows the distribution of the KAM values for all points on the EBSD maps. The distributions from all experimental steels were identical.

**Figure 8:**
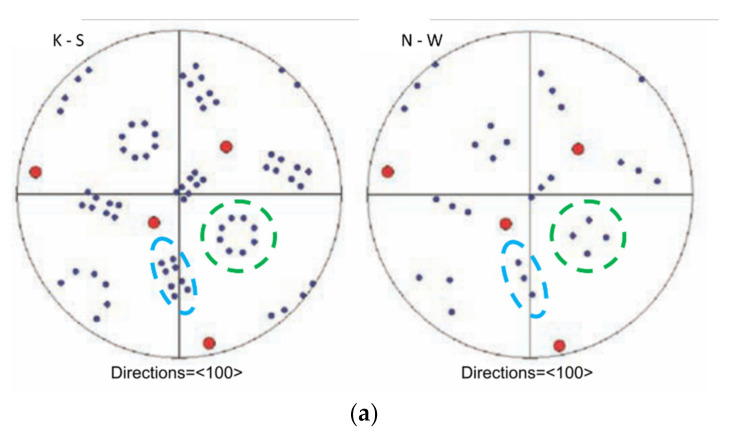

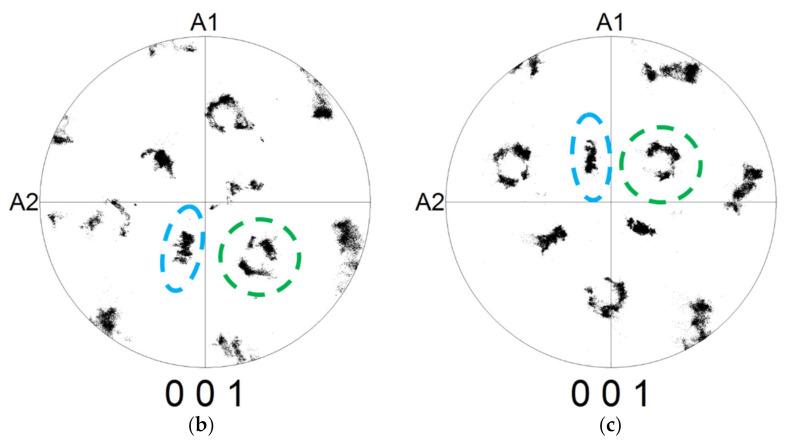
Pole figures (PFs) of ferrite crystals from one prior austenite grain. (**a**) Modeled PF according to K-S and N-W orientation relationships (red dots represents directions of the original austenite lattice, blue and green lines encircle characteristic features in the PF) source [24]; (**b**) PF from one prior austenite grain in the 0Cu 15-min treated sample; (**c**) PF from one prior austenite grain in the 1.5Cu 15-min treated sample. Reproduced with permission from ref. [24]. Copyright 2011 The Chinese Society for Metals. Published by Elsevier Ltd.

Pole figures (PFs) for single prior austenite grains were obtained to determine the orientation relationship between austenite and ferrite during bainitic transformation. Figure 8a shows the modeled PF for the Kurdjumow–Sachs (K-S) and Nishiyama–Wassermann (N-W) orientation relationships [24]. The modeled PFs both look similar with individual orientation variants (points) arranged in two types of repeating groups, i.e., an elongated set of eight (K-S) or three (N-W) variants and a group of eight (K-S) resp. 4 (N-W) of octagonal resp. square arrangement. These groups are highlighted in the modeled PF. Corresponding groups of variants are also highlighted in the measured PF in Figure 8b,c. It seems that the shapes of these groups match with the PF of the K-S orientation relationship. The same appearance was observed for the PF of all experimental steels.

The EBSD analysis proved that different Cu and Mn contents did not have any observable impact on the bainitic ferrite morphology, grain size, dislocation density, or the bainitic transformation mechanism. Therefore, these factors contributed to the YS by the same grade for all experimental steels and cannot explain the differences among the YS values of individual steels.

The remaining strengthening factors are solid solution and precipitation strengthening. The bainitic transformation is a shear diffusionless transformation for iron and substitutional alloying atoms. Carbon diffusion proceeds during the bainitic transformation [22] and in this experiment, led to carbide precipitation. The carbides strengthen the ferrite crystals, and their strengthening effect is given by their size, spatial density, and shape. The high-resolution SEM micrographs allowed limited comparison of carbide size and shape. The disc or rod-like carbides grow in a specific direction in lower bainite crystals, which requires a particular orientation of the sectioned bainite crystal in order to reveal them as parallel elongated particles. Etching depth also influences the apparent size and density of the carbides, however, only a qualitative assessment could be drawn. Carbides can be observed as elongated parallel particles that seem to have a similar size and shape in all experimental steels. It can be tentatively speculated that all bainitic crystals have similar sized and spaced carbides in their interior, even if their orientation relative to the metallography section does not allow clear observation of these features. Apparent carbide size, shape, and distribution did not change from 15- to 120-min holding treatments at 400 °C. This was confirmed by the fact that the YS and UTS values of the 0Cu steel did not change significantly between the 15- and 120-min treated samples.

Precipitation strengthening can also be achieved using Cu. It is reasonable to expect no Cu precipitation after the 15-min holding treatment. The bainitic transformation is diffusionless for Cu, therefore, it cannot precipitate during the transformation itself. The 15-min treatment at 400 °C is not a sufficient time for noticeable Cu precipitation according to the literature [16,17,18] and a previous study of martensite tempering [21]. Therefore, differences in YS and UTS values among experimental steels can be explained as a solid solution strengthening by Cu.

**Figure 9:**
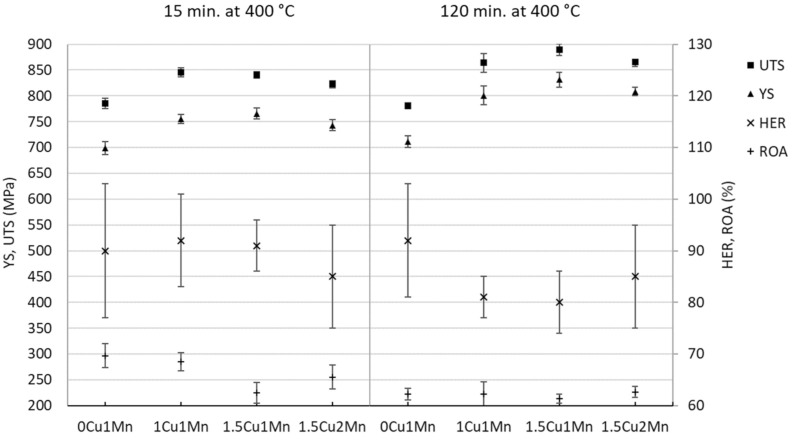
Comparison of YS, UTS, hole expansion ratio (HER), and reduction of area (ROA) values for all tested material states.

Figure 9 compares the YS, UTS, ROA, and HER values of the experimental steels after 15 and 120 min of holding treatments at 400 °C. The 15-min treatment caused roughly a 50 MPa increase in the YS and UTS values of the Cu-alloyed steels as compared with the 0Cu steel. This strengthening was not dependent on the Cu and Mn content as solid-solution strengthening should be. This fact might be caused by slight carbide precipitation differences among experimental steels which were not apparent from the SEM micrographs.

The 120-min holding treatment at 400 °C caused significant strengthening of Cu-alloyed steels, which can be attributed to the Cu precipitation. The precipitates’ presence is documented in Figure 6. The 1Cu and 1.5Cu steels had higher YS values by 90 and 120 MPa, respectively, as compared with the 0Cu steel, which was comparable with a previous study [21] where the same experimental steels underwent quenching and tempering. The 1Cu and 1.5Cu steels differed from the 0Cu YS by 57 MPa and 145 MPa after 120-min tempering at 400 °C.

The Mn content enhancement from 1 to 2 wt.% did not boost the Cu strengthening ability. The 1.5Cu2Mn steel showed even lower strength than the 1.5Cu steel. This is in contrast with the martensite tempering approach where 1.5Cu2Mn steel exhibited the highest YS after 2 h tempering at 400 °C (189 MPa higher than YS of 0Cu steel). The Mn and Cu exhibited a synergy effect for Cu precipitation strengthening during martensite tempering but not during the bainite isothermal holding treatment at the same temperature of 400 °C.

Mn was observed as an active element in Cu precipitation forming Mn-enriched shells at the Cu-Fe interphase [19]. It has been proposed that these shells lower specific interphase energy of the precipitate [20]. Mn could, therefore, influence the Cu precipitation kinetics and subsequent precipitate coarsening. On the one hand, this influence has indeed been observed during martensite tempering [21]; steels with enhanced Mn content exhibited higher values of Cu precipitation strengthening. On the other hand, this effect was not observed in experiments in this study with bainitization and subsequent isothermal holding.

The cause of this difference has to be found in the different microstructures in which Cu precipitation occurs. Lower bainite seemed to be unaffected by 400 °C hold prolongation from 15 to 120 min. The mechanical properties as well as the microstructure appearance were the same. There were no signs of carbide coarsening or ferrite crystals recovery found during the 15 to 120 min interval at 400 °C. Therefore, only Cu precipitation took place in the Cu-alloyed steels. The enhancement of Mn content from 1 to 2 wt.% did not promoted Cu strengthening ability in this case.

Martensite tempering is a very complex process. Several phenomena take place simultaneously during the tempering-carbon diffusion, carbide precipitation, and ferrite lattice recovery (vacancy and dislocation density reduction) [25]. Cu precipitation proceeds on top of these phenomena. Enhancing of the Mn content from 1 to 2 wt.% promoted Cu strengthening ability, in this case significantly. Thus, influence of Mn on Cu precipitation seems to be bound to the complexity of martensite tempering rather than to Cu precipitation itself.

The toughness of the experimental steels can be measured as an area under the stress-strain curve. Toughness itself is a work (measured in J) necessary for dividing material in two pieces. The stress-strain curve is a dependence of stress in MPa on the dimensionless elongation, giving the area under curve (AuC, see Table 3) in MPa. This can be interpreted as a specific toughness, work per volume (J per m^3^ gives Pa in the terms of units) determined from the whole measured volume of the sample. An increase in YS and UTS by Cu precipitation enhanced the toughness of the samples. It is expected, because the overall shape of the stress-strain curve did not change with chemical composition or bainitization duration and the ductility A_80_ was also comparable for all samples. The AuC just increased as the stress-strain curves reached higher stress levels.

Bainitization for 120 min. did not cause a decrease in ductility, but a decrease in the ROA was clearly visible (as compared with 15-min bainitization). The ROA was similar for all experimental steels and generally lower than for the 15-min treated samples. This implies that other phenomena than Cu precipitation was responsible for the decrease in the ROA after log duration holding at 400 °C. This is a motivation for further detailed TEM analysis of the bainite structure to reveal the cause.

The 120-min treated samples had a trend of inversely dependent HER and YS values (or UTS). However, there was quite a large scattering of HER values which weakened the confidence in this dependence.

The experimental steels generally exhibited good values of HER. They surpassed the values of HER typical for dual steels of comparable UTS [1,3]. This can be attributed to favorable fully bainitic structure. Poor edge stretchability of dual phase steels is explained as a result of having soft (ferrite) and hard (martensite) phases mixed in the microstructure. From this point of view, monophase steels are favorable for applications requiring good edge stretchability. The HER values obtained in this study are comparable with other studies of fully martensitic or bainitic steels.

Cu precipitation strengthening itself did not seem to cause a reduction in steel ductility A80, however it was correlated with a reduction in the HER. The strength-plasticity trade-off was not demonstrated in pair “strength-ductility”, but in pair “strength-edge stretchability”.

## 5. Conclusions

Bainitization with subsequent holding at bainitization temperature proved to be a feasible procedure to induce Cu precipitation and strengthening of steel sheet. This procedure can be readily applicable into steelmaking processes of controlled hot rolling with subsequent coiling in a “warm” state. Cu precipitation introduced a considerable increase in both YS (119 MPa for 1.5% Cu) and UTS values (109 MPa for 1.5% Cu) and caused a reduction in edge stretchability by 12%. The Mn content enhancement did not boost the Cu-strengthening ability. The Mn-Cu precipitation synergy clearly depends on other processes occurring during the martensite tempering and it is worthwhile to investigate this phenomenon in detail with high-resolution techniques.

## Figures and Tables

**Table 1:** Chemical compositions of the experimental steels. All concentrations in wt. %.

Material	C	Cu	Mn	Si	Ti	B	N
0Cu	0.22	0.12	0.98	0.07	0.022	0.0014	0.0056
1Cu	0.21	1.08	0.98	0.08	0.025	0.0013	0.0063
1.5Cu	0.21	1.49	0.99	0.10	0.022	0.0013	0.0054
1.5Cu2Mn	0.21	1.49	2.00	0.08	0.023	0.0015	0.0057

**Table 2:** Results of the tensile test and HET for 15- and 120-min bainitization treatments.

15 min	YS (MPa)	UTS (MPa)	A_g_ (%)	A_80_ (%)	ROA (%)	HER (%)
0Cu	698 ± 13	785 ± 10	3.6 ± 0.2	7.7 ± 0.2	70 ± 2	90 ± 13 *
1Cu	755 ± 8	845 ± 8	4.3 ± 0.2	8.0 ± 0.5	68 ± 2	92 ± 9
1.5Cu	765 ± 11	840 ± 6	3.9 ± 0.4	7.5 ± 0.4	62 ± 2	91 ± 5
1.5Cu2Mn	743 ± 11	823 ± 7	4.1 ± 0.2	8.2 ± 0.2	66 ± 2	85 ± 10
**120 min**	**YS (MPa)**	**UTS (MPa)**	**A_g_ (%)**	**A_80_ (%)**	**ROA (%)**	**HER (%)**
0Cu	712 ± 11	781 ± 5	4.7 ± 0.9	8.0 ± 1.1	62 ± 1	92 ± 11 *
1Cu	801 ± 18	864 ± 18	4.7 ± 0.8	8.5 ± 0.6	62 ± 2	81 ± 4
1.5Cu	831 ± 15	890 ± 11	5.0 ± 0.3	8.0 ± 1.0	61 ± 1	80 ± 6
1.5Cu2Mn	808 ± 8	865 ± 8	4.7 ± 0.3	8.5 ± 0.3	63 ± 1	85 ± 10

* as a result of 5 tested specimens.

**Table 3:** Yield stress (YS) and ultimate tensile stress (UTS) values as compared with values of engineering stress drop at fracture from the maximal value (decreased UTS) and the area under the stress-strain curve (AuC) obtained from the stress-strain curves analysis.

15 min	YS (MPa)	UTS (MPa)	UTS Drop (%)	AuC (MPa)
0Cu	698 ± 13	785 ± 10	30 ± 2	49 ± 2
1Cu	755 ± 8	845 ± 8	32 ± 1	58 ± 2
1.5Cu	765 ± 11	840 ± 6	26 ± 3	54 ± 3
1.5Cu2Mn	743 ± 11	823 ± 7	29 ± 1	57 ± 2
**120 min**	**YS (MPa)**	**UTS (MPa)**	**UTS drop (%)**	**AuC (MPa)**
0Cu	712 ± 11	781 ± 5	32 ± 2	51 ± 6
1Cu	801 ± 18	864 ± 18	34 ± 4	60 ± 8
1.5Cu	831 ± 15	890 ± 11	28 ± 6	64 ± 6
1.5Cu2Mn	808 ± 8	865 ± 8	25 ± 3	64 ± 3

**Table 4:** Grain sizes for 15-min treated samples. Grains were defined as areas enclosed by boundaries with >2° misorientation.

Sample	Average Grain Size (µm)
0Cu	1.3
1Cu	1.2
1.5Cu	1.3
1.5Cu2Mn	1.3

## Data Availability

The raw data are not publicly available due to ongoing research.
